# Impact of gender and side of surgery on cognition, affectivity, and quality of life in patients undergoing temporal lobe epilepsy surgery: a prospective cohort study

**DOI:** 10.1186/s13293-025-00775-8

**Published:** 2025-11-05

**Authors:** Irene Cano-López, Judit Catalán-Aguilar, Kevin G. Hampel, Alejandro Lozano-García, Paula Tormos-Pons, Esperanza González-Bono, Vicente Villanueva

**Affiliations:** 1 Institut d’Investigació en Psicologia dels Recursos Humans, del Desenvolupament Organitzacional i de la Qualitat de Vida Laboral (IDOCAL), Department of Psychobiology, Psychology Center, Universitat de València, Valencia, Spain; 2 Refractory Epilepsy Unit, Neurology Service, Member of ERN EPICARE, Hospital Universitario y Politécnico La Fe, Valencia, Spain; 3 Faculty of Health Sciences, Valencian International University, Valencia, Spain; 4 Department of Psychology, Universidad Europea de Valencia, Valencia, Spain

**Keywords:** Lateralization, Neuropsychology, Gender differences, Psychosocial functioning, Temporal lobectomy, Seizure

## Abstract

**Background:**

To examine the impact of gender and its interaction with the side of surgery on cognition, affectivity, and quality of life (QOL) in drug-resistant epilepsy, as well as postsurgical changes in these domains.

**Methods:**

In this prospective cohort study, 86 adults with TLE (46 women and 40 men) underwent a neuropsychological evaluation before and one year after surgery, including attention, executive function, language, verbal and visual memory, anxiety, depression, and QOL outcomes.

**Results:**

After surgery, 84.1% of patients were seizure-free. In the group with right-sided surgery, men had poorer executive functioning (*p* = 0.05) and memory than women (for all, *p* < 0.05), independently of the time point (i.e., before or after surgery). Men with right-side surgery showed poorer executive function than those with left-side surgery (for all, *p* < 0.04), and a postsurgical anxiety decrease (*p* < 0.001). Women with right-side surgery had a better memory than those with left-side surgery, independently of the time point (for all, *p* < 0.001). Both genders showed postsurgical QOL improvements modulated by surgery side (*p* = 0.037). Regardless of the surgery side, women had poorer semantic fluency (*p* = 0.03) and QOL (*p* = 0.05) than men and postsurgical semantic fluency declines (*p* = 0.024), whereas men had postsurgical executive function declines (*p* = 0.05).

**Conclusions:**

These findings underscore the importance of accounting for both gender and the side of surgery in understanding cognitive, affective, and QOL outcomes in patients with TLE, and could be useful for designing targeted neuropsychological interventions.

## Plain language summary

Temporal lobe epilepsy (TLE) is a condition that involves seizures and other challenges. Surgery is often used to help control seizures and improve the quality of life, but it can also affect cognitive functions. Women with TLE might experience different changes compared to men, and these changes may depend on which side of the brain is operated on. This study looked at how surgery affects the brain and overall well-being in 86 adults (46 women and 40 men) with TLE. We focused on how gender and the side of the surgery (left or right) influenced cognitive functions, affectivity, and quality of life. The patients were tested before surgery and again one year later. The results showed that most patients (84%) were seizure-free after surgery. Women who had surgery on the right side of their brain performed better on memory and executive functions compared to men. Also, women who had right-sided surgery had better memory than those who had left-sided surgery. Men with right-side surgery had poorer executive function than those with left-side surgery, and showed a decrease in anxiety after surgery. Both men and women showed improvements in quality of life after surgery, which were influenced by which side of the brain was operated on. Independently of the side of the surgery, women experienced a decline in word fluency, while men showed declines in executive function. These findings suggest that understanding how gender and the side of surgery interact can help healthcare professionals better plan treatments for patients with TLE.

## Highlights

In the right-sided surgery group, men had poorer executive function and memory than women.

Men with right-side surgery showed poorer executive function than those with left-side surgery.

Men with right-side surgery had a postsurgical anxiety decrease.

Women with right-side surgery had a better memory than those with left-side surgery.

Quality of life improved after surgery in both genders, modulated by surgery side.

## Background

Patients with temporal lobe epilepsy (TLE) exhibit considerable variability in their cognitive profiles [1] and post-surgical outcomes [2]. While surgery is generally associated with improvements in affectivity and quality of life (QOL) [3], it can also lead to cognitive side effects [4], potentially influencing these outcomes. Factors such as gender and the side of seizure focus may modulate this variability and interact with each other.

Although gender is frequently reported in research, it is often used only descriptively or as a control variable, with few studies conducting direct gender-based comparisons. These studies have found gender differences in memory [5, 6] and affectivity [7]. Furthermore, epilepsy is not experienced equally by men and women, as it involves socially marked behaviors associated with gender roles, which may influence QOL [8].

Independently of gender, the side of seizure focus has been recognized as a key determinant of cognitive outcomes. Compared to patients with right TLE, patients with left TLE have poorer verbal memory [9] and greater risk of post-surgical verbal memory and naming impairments [4]. Beyond memory and naming, the nociferous cortex hypothesis suggests that, in TLE, the epileptogenic cortex may disrupt extratemporal regions involved in executive processes, thereby contributing to performance deficits, whereas successful TLE surgery may facilitate improvements in executive functioning [10]. Nevertheless, the role of the side of seizure focus in attention and executive functions has been scarcely explored, yielding inconclusive findings [11–14], and its impact on post-surgical changes in these functions remains unclear [2].

The interaction between gender and side of seizure focus may significantly influence cognitive, affective, and QOL outcomes. However, research examining this interaction is limited, focusing mainly on gender differences in memory and without considering affectivity and QOL. Studies suggest that women have better verbal memory performance than men [15, 16], and others have reported a greater vulnerability to verbal memory decline in men after left TLE surgery, indicating greater verbal memory plasticity in women [17, 18]. To date, only Baxendale et al. [19] have examined cognitive and affective variables together, finding that women demonstrate better presurgical verbal memory – despite poorer naming ability – but experience greater post-surgical decline than men, with no significant differences in depression or anxiety. While this study represents an important advance, it did not explore potential gender differences in attention, executive functions, and QOL.

Based on the above, our study aimed to examine the impact of gender and its interaction with the side of surgery on cognitive functioning (i.e., attention, executive function, language, verbal and visual memory), affectivity, and QOL in patients with drug-resistant TLE – as well as assessing changes in these variables following surgery.

## Methods

### Participants

Participants were recruited from the Refractory Epilepsy Unit at the Hospital Universitario y Politécnico La Fe (Valencia, Spain), a member of the European Reference Network for Epilepsy (EpiCARE), between April 2015 and September 2024. This study included patients who met the following criteria: (a) a diagnosis of drug-resistant TLE who underwent resective epilepsy surgery restricted to the temporal lobe; (b) aged 18 years or more; (c) having undergone a neuropsychological assessment performed before surgery and 12 months after surgery. Exclusion criteria were: (a) seizure onset zone involving extratemporal regions (e.g., frontotemporal epilepsy); (b) not having completed primary education; (c) aged over 65; (d) inability to undergo reliable assessment due to severe cognitive impairment; (e) history of severe psychiatric conditions; or (f) lack of fluency in Spanish.

### Procedure

This prospective cohort study adhered to the Strengthening the Reporting of Observational Studies in Epidemiology (STROBE) statement guidelines [20]. The study protocol complied with the principles of the Declaration of Helsinki and received approval from the Ethics Committee of Hospital Universitario y Politécnico La Fe. Informed consent was obtained from all participants.

A multidisciplinary team carried out a presurgical assessment, including seizure history and semiology, neurological evaluation, prolonged video-electroencephalography monitoring, 3-Tesla magnetic resonance imaging, psychiatric evaluation, and neuropsychological evaluation. Selective additional assessments, including fluorodeoxyglucose-positron emission tomography, single-photon emission computed tomography, and intracranial EEG recording, were performed as needed. This comprehensive approach enabled accurate diagnosis of epilepsy type and localization of the epileptogenic area. Demographic data along with clinical variables were recorded. The ASM drug load was calculated based on the defined daily dose (DDD), representing the “assumed average maintenance dose per day” [21]. Each patient’s daily dose for a specific ASM was divided by its corresponding DDD to compute the dose ratio (daily dose/DDD). The total ASM drug load was obtained by summing the dose ratios of all ASMs taken by each patient [22].

Surgery was carried out after the presurgical assessment and consisted of a temporal lobectomy, temporal lobectomy with amygdalohippocampectomy, or temporal lobe lesionectomy targeting circumscribed lesions, depending on the case. The majority of lesionectomies involved the lateral part of the temporal lobe or the temporal pole; however, in a minority of patients, the circumscribed lesions were located within mesial temporal structures, and resections were adapted accordingly. The neuropsychological evaluation was performed before surgery and 12 months after surgery. Both evaluations were similar, except for the intelligence quotient (IQ), which was only assessed in the presurgical evaluation. Post-surgical seizure outcome was assessed by a neurologist using the Engel Epilepsy Surgery Outcome Scale [23].

### Neuropsychological assessment

The *Wechsler Abbreviated Scale of Intelligence* (WASI-II) [24] was used to assess IQ (test-retest reliability: 0.90–0.96). Four subtests (vocabulary, similarities, block design, and matrix reasoning) were combined to form the full-scale IQ score, with higher scores indicating better IQ.

The *EpiTrack* [25] was used to assess response inhibition, attention, cognitive tracking, processing speed, verbal fluency, and working memory (test-retest reliability: 0.79). A total age-adjusted EpiTrack score was calculated, ranging from 9 to 49 points, with higher scores indicating better performance.

*The Wisconsin Card Sorting Test* (WCST) [26] was employed to assess cognitive flexibility, abstract conceptualization, and responsiveness to feedback (reliability: 0.83). Higher scores indicated poorer performance in the following indices: number of total errors, non-perseverative errors, and perseverative responses; trials to complete the first category; and failure to maintain a set. Additionally, higher scores showed better performance in the following indices: correct responses; conceptual level responses; categories completed; and learning to learn. All the above variables were considered to capture a comprehensive profile of executive functioning, as they reflect distinct cognitive processes such as cognitive flexibility, set shifting, and perseveration.

The *Boston Naming Test* (BNT) [27] was used to assess visual confrontation naming (test-retest reliability: 0.86). The total score was computed as the number of cards correctly named without phonemic cues (with 60 being the maximum score).

*FAS* [28] was employed to evaluate phonemic fluency (test-retest reliability: 0.83). Participants were requested to say as many words as they could starting with the letters F, A, and S in 1 min. We computed the sum of all admissible words for the three letters (i.e., total score).

The *Animal Naming Test* [29] was used to assess semantic fluency (test-retest reliability: 0.56). Participants were asked to say as many animals as possible in one minute. We computed a total score based on the sum of admissible words for this semantic category.

*Wechsler Memory Scale-Third Edition* (WMS-III) [30, 31] was used to assess verbal and visual memory (test-retest reliability: 0.87). Two general memory indices (immediate and delayed memory, combining verbal and visual information), and material-specific memory indices (immediate auditory memory, delayed auditory memory, delayed auditory recognition, immediate visual memory, and delayed visual memory) were computed and presented in age-adjusted percentile scores. Verbal memory indices were computed based on Logical Memory and Verbal Paired Associates subtests, while visual memory indices were calculated based on Faces and Family Pictures subtests.

The *Trait Anxiety Scale of the State-Trait Anxiety Inventory* (STAI) [32, 33] evaluates relatively stable aspects of anxiety and is composed of 20 items rated on a four-point scale ranging from 0 (“hardly never”) to 3 (“almost always”), with higher scores indicating higher anxiety (Cronbach’s alpha: 0.94).

The *Beck Depression Inventory-II* (BDI-II) [34] evaluates depression through 21 items rated on a four-point scale (Cronbach’s alpha: 0.89). We computed the total score, with higher scores indicating higher depression levels.

The *Quality of Life in Epilepsy Inventory* (QOLIE-31) [35, 36] was used to evaluate QOL (Cronbach’s alpha: 0.92). It includes 31 items across seven domains: seizure worry, overall QOL, emotional well-being, energy, cognitive self-assessment, medication effects, and social functioning. Domain scores were converted to a 0-100 scale, with higher scores indicating better QOL. A composite QOL score was calculated as a weighted average of the domain scores.

### Statistical analysis

The Kolmogorov-Smirnov test was carried out to examine the normality of the data. ANOVAs were conducted for between-group comparisons of continuous demographic and clinical variables based on gender and side of surgery, while the chi-square test was used to study the differences in categorical variables.

Postsurgical changes in cognition, affectivity, and QOL were described using the reliable change index (RCI) [37]. Due to the absence of a control group in our study and the lack of specific normative data for individuals with epilepsy, RCIs were derived from the SD and reliability data for each test obtained from the general population [4, 38, 39], except for the total score of EpiTrack, which directly provides RCIs for people with epilepsy.

To explore differences in IQ (pre-surgery) depending on gender and side of surgery, ANCOVAs were conducted with “gender” and “side of surgery” as between-participant factors, and total ASM drug load as a covariate. Repeated-measures ANCOVAs were used to analyze differences in cognitive domains, affectivity, and QOL, with “gender” and “side of surgery” as between-participant factors, “time point” (pre- vs. post-surgery) as the within-participant factor, and total ASM drug load as a covariate. Greenhouse-Geisser adjustments were applied to provide a conservative correction that reduces the risk of Type I error in the overall ANOVA, and Bonferroni tests were used as post hoc analyses to control for Type I error across multiple comparisons. Effect sizes were calculated using the partial eta squared (η^2^) statistic, with thresholds of 0.01 (small), 0.06 (medium), and 0.14 (large) [40].

These analyses were performed using SPSS 28.0. Two-tailed tests with *p* set at 0.05 were considered significant. No missing data were detected.

## Results

### Characteristics of the participants

The sample comprised 86 adult patients with drug-resistant TLE (mean age = 39.83 years, SD = 12.55): 26 women and 18 men underwent left-sided surgery, while 20 women and 22 men underwent right-sided surgery (Table 1). Post-surgical assessment was conducted on average 12 months after surgery. There were no losses to follow-up.

**Table 1:** Participants’ characteristics depending on gender and side of surgery (mean ± SD or n (%))

Characteristics	Total (*N* = 86)	Women left-sided surgery (*n* = 26)	Men left-sided surgery (*n* = 18)	Women right-sided surgery (*n* = 20)	Men right-sided surgery (*n* = 22)	*p*-value
Age (years)	39.83 ± 12.55	42.12 ± 11.95	36.78 ± 11.34	40.30 ± 13.67	39.18 ± 13.37	G: 0.24 S: 0.92 G×S: 0.45
Educational level						G: 0.48 S: 0.88 G×S: 0.93
Primary	11 (12.8%)	3 (11.5%)	3 (16.7%)	2 (10.0%)	3 (13.6%)	
Secondary	46 (53.5%)	14 (53.8%)	9 (50.0%)	11 (55.0%)	12 (54.5%)	
Undergrad university	12 (14.0%)	2 (7.7%)	3 (16.7%)	3 (15.0%)	4 (18.2%)	
Postgrad university	17 (19.8%)	7 (26.9%)	3 (16.7%)	4 (20.0%)	3 (13.6%)	
Marital status						G: 0.33 S: 0.31 G×S: 0.25
Single	42 (48.8%)	10 (38.5%)	9 (50.0%)	9 (45.0%)	14 (63.6%)	
Married	39 (45.3%)	14 (53.8%)	7 (38.9%)	10 (50.0%)	8 (36.4%)	
Divorced	5 (5.8%)	2 (7.7%)	2 (11.1%)	1 (5.0%)	0 (0.0%)	
Age at epilepsy onset (years)	18.64 ± 12.94	17.92 ± 12.76	20.17 ± 13.00	18.59 ± 13.15	18.27 ± 13.72	G: 0.74 S: 0.83 G×S: 0.66
Epilepsy duration (years)	21.19 ± 15.62	24.19 ± 15.34	16.61 ± 17.64	21.72 ± 16.27	20.91 ± 13.68	G: 0.22 S: 0.79 G×S: 0.32
Presurgical seizure frequency (per month)	9.69 ± 17.13	8.56 ± 14.67	14.44 ± 31.82	9.35 ± 8.52	7.67 ± 7.49	G: 0.58 S: 0.43 G×S: 0.32
Seizure type						G: 0.51 S: 0.13 G×S: 0.16
FPC	3 (3.5%)	1 (3.8%)	1 (5.6%)	0 (0.0%)	1 (4.5%)	
FIC	31 (36.0%)	8 (30.8%)	2 (11.1%)	12 (60.0%)	9 (40.9%)	
FBTC	1 (1.2%)	0 (0.0%)	1 (2.5%)	0 (0.0%)	0 (0.0%)	
FPC + FIC	18 (20.9%)	8 (30.8%)	3 (16.7%)	2 (10.0%)	5 (22.7%)	
FPC + FBTC	1 (1.2%)	0 (0.0%)	0 (0.0%)	0 (0.0%)	1 (4.5%)	
FIC + FBTC	27 (31.4%)	7 (26.9%)	9 (50.0%)	5 (25.0%)	6 (27.3%)	
FPC + FIC + FBTC	5 (5.8%)	2 (7.7%)	2 (11.1%)	1 (5.0%)	0 (0.0%)	
MRI findings						G: 0.72 S: 0.54 G×S: 0.29
HS	38 (44.2%)	15 (57.7%)	6 (33.3%)	8 (40.0%)	9 (40.9%)	
Focal cortical dysplasia	7 (8.1%)	2 (7.7%)	2 (11.1%)	2 (10.0%)	1 (4.5%)	
Gliosis	2 (2.3%)	0 (0.0%)	1 (5.6%)	1 (5.0%)	0 (0.0%)	
Tumor	13 (15.1%)	4 (15.4%)	3 (16.7%)	3 (15.0%)	3 (13.6%)	
Heterotopia	4 (4.7%)	1 (3.8%)	0 (0.0%)	1 (5.0%)	2 (9.1%)	
Cavernoma	8 (9.3%)	2 (7.7%)	4 (22.2%)	1 (5.0%)	1 (4.5%)	
Atrophy	1 (1.2%)	0 (0.0%)	0 (0.0%)	1 (5.0%)	0 (0.0%)	
Encephalomalacia	1 (1.2%)	0 (0.0%)	0 (0.0%)	1 (5.0%)	0 (0.0%)	
Non-specific pathology	12 (14.0%)	2 (7.7%)	2 (11.1%)	2 (10.0%)	6 (27.3%)	
HS						G: 0.24 S: 0.50 G×S: 0.39
Yes	38 (44.2%)	15 (57.7%)	6 (33.3%)	8 (40.0%)	9 (40.9%)	
No	48 (55.8%)	11 (42.3%)	12 (66.7%)	12 (60.0%)	13 (59.1%)	
Number of ASMs	2.70 ± 0.83	2.35 ± 0.69	2.72 ± 0.96	2.85 ± 0.93	2.95 ± 0.65	G: 0.17 S: 0.038* G×S: 0.44
Number of failed ASMs	6.13 ± 3.51	5.77 ± 2.90	6.00 ± 4.54	6.55 ± 3.65	6.27 ± 3.33	G: 0.98 S: 0.50 G×S: 0.75
Total ASM drug load	3.11 ± 1.32	2.81 ± 1.16	3.27 ± 1.45	2.86 ± 1.59	3.54 ± 1.06	G: 0.05* S: 0.58 G×S: 0.71
Surgical approach						G: 0.24 S: 0.60 G×S: 0.16
TL	8 (9.3%)	4 (15.4%)	1 (5.6%)	0 (0.0%)	3 (13.6%)	
TL + AH	55 (64.0%)	18 (69.2%)	8 (44.4%)	15 (75.0%)	14 (63.6%)	
Temporal lobe lesionectomy	23 (26.7%)	4 (15.4%)	9 (50.0%)	5 (25.0%)	5 (22.7%)	
Engel I						G: 0.76 S: 0.09 G×S: 0.36
Yes	70 (81.4%)	24 (92.3%)	15 (83.3%)	14 (70.0%)	17 (77.3%)	
No	16 (18.6%)	2 (7.7%)	3 (16.7%)	6 (30.0%)	5 (22.7%)	

Note. AH: amygdalohippocampectomy; ASMs: anti-seizure medications; FBTC: focal-to-bilateral tonic-clonic seizure; FIC: focal impaired consciousness seizure; FPC: focal preserved consciousness seizure; G: gender effects; G×S: gender×side of surgery effects; HS: hippocampal sclerosis; MRI: magnetic resonance imaging; S: side of surgery effects; TL: temporal lobectomy; *: *p* < 0.05

Men consumed a higher total ASM drug load than women (*F*[1,85] = 3.98, *p* = 0.05, η² = 0.05), and right-sided surgery patients consumed a higher number of ASMs than left-sided surgery patients (*F*[1,85] = 4.43, *p* = 0.038, η² = 0.05). Given the strong correlation between ASM drug load and the number of ASMs (*r* = 0.76, *p* < 0.001), total ASM drug load was included as a covariate in further analyses to avoid multicollinearity, as it provides a more precise measure of pharmacological load. No other significant demographic or clinical differences, such as the presence of hippocampal sclerosis or the surgical approach, were found based on gender, surgery side, or their interaction.

Considering RCI, reliable postsurgical changes in cognitive, affective, and QOL variables in the total sample are shown in Table 2.

**Table 2:** N (%) of patients with improvements, no changes and declines in each test

Variables	Reliability	SD	RCI criterion	Improvement	No change	Decline
Epitrack [25]	0.90					
Interference test		4.10	± 3.59	16 (18.6%)	62 (72.1%)	8 (9.3%)
TMT A		10.40	± 9.12	13 (15.1%)	54 (62.8%)	19 (22.1%)
TMT B		28.20	± 24.72	14 (16.1%)	59 (68.6%)	13 (15.1%)
Maze test		16.10	± 14.11	11 (12.8%)	67 (77.9%)	8 (9.3%)
Word fluency		6.80	± 5.96	24 (27.9%)	53 (61.6%)	9 (10.5%)
Digit span backward		1.30	± 1.14	8 (9.3%)	70 (81.4%)	8 (9.3%)
Total score		3.30	≥ 4: improvement -2 to 3: no change ≤ -3: decline	11 (12.8%)	62 (72.1%)	13 (15.1%)
WCST [26]	0.83					
Number of errors		20.01	± 22.87	6 (7.0%)	72 (83.7%)	8 (9.3%)
Number of perseverative responses		15.25	± 17.43	10 (11.6%)	65 (75.6%)	9 (10.5%)
Number of non-perseverative errors		9.66	± 11.04	6 (7.0%)	64 (74.4%)	16 (18.6%)
Trials to complete the first category		14.93	± 17.06	6 (7.0%)	69 (80.2%)	11 (12.8%)
Failure to maintain a set		1.01	± 1.15	8 (9.3%)	73 (84.9%)	5 (5.8%)
Correct responses		10.85	± 12.40	5 (5.8%)	66 (76.7%)	15 (17.4%)
Conceptual level responses		20.08	± 22.95	6 (7.0%)	70 (81.4%)	10 (11.6%)
Categories completed		1.52	± 1.74	5 (5.8%)	66 (76.7%)	15 (17.4%)
Learning to learn		5.64	± 6.45	10 (11.6%)	51 (59.3%)	16 (18.6%)
BNT [27]	0.86	3.47	± 3.60	8 (9.3%)	54 (62.8%)	24 (27.9%)
FAS [28]	0.83	13.10	± 14.97	1 (1.2%)	84 (97.7%)	1 (1.2%)
Animal Naming Test [29]	0.56	5.00	± 9.19	2 (2.3%)	82 (95.3%)	2 (2.3%)
WMS-III [30, 31]	0.87					
Immediate auditory memory		13.70	± 13.69	1 (1.2%)	85 (98.8%)	0 (0.0%)
Immediate visual memory		14.70	± 14.69	0 (0.0%)	86 (100.0%)	0 (0.0%)
Immediate memory		14.50	± 14.49	1 (1.2%)	83 (96.5%)	2 (2.3%)
Delayed auditory memory		14.10	± 14.09	1 (1.2%)	85 (98.8%)	0 (0.0%)
Delayed visual memory		14.70	± 14.69	0 (0.0%)	86 (100.0%)	0 (0.0%)
Delayed memory		14.60	± 14.59	3 (3.5%)	74 (86.0%)	9 (10.5%)
Delayed auditory recognition		14.40	± 14.39	0 (0.0%)	86 (100.0%)	0 (0.0%)
STAI [32, 33]	0.89	10.39	± 9.55	22 (25.6%)	54 (62.8%)	10 (11.6%)
BDI-II [34]	0.87	17.90	± 17.89	4 (4.7%)	77 (89.5%)	5 (5.8%)
QOLIE-31 [35, 36]	0.92					
Seizure worry		26.00	± 20.38	39 (45.3%)	41 (47.7%)	6 (7.0%)
Overall QOL		18.00	± 14.11	33 (38.4%)	45 (52.3%)	8 (9.3%)
Emotional well-being		19.00	± 14.90	31 (36.0%)	41 (47.7%)	14 (16.3%)
Energy		21.00	± 16.46	21 (24.4%)	51 (59.3%)	14 (16.3%)
Cognition self-rating		23.00	± 18.03	33 (38.4%)	41 (47.7%)	12 (14.0%)
Medication effects		31.00	± 24.30	31 (36.0%)	44 (51.2%)	11 (12.8%)
Social functioning		27.00	± 21.17	31 (36.0%)	47 (54.7%)	8 (9.3%)
QOLIE-31 total score		16.00	± 12.54	36 (41.9%)	39 (45.3%)	11 (12.8%)

Note: BDI: Beck Depression Inventory-II; BNT: Boston Naming Test; QOL: quality of life; QOLIE-31: Quality of Life in Epilepsy Inventory; STAI: State-Trait Anxiety Inventory; TMT: Trail Making Test; WCST: Wisconsin Card Sorting Test; WMS-III: Wechsler Memory Scale-Third Edition

### Gender and side of surgery effects on cognitive functioning, affectivity, and QOL and postsurgical changes

Neuropsychological scores are shown in Table 3.

**Table 3:** Scores in neuropsychological tests depending on gender and side of surgery (mean ± SD)

Variables	Women left-sided surgery (*n* = 26)		Men left-sided surgery (*n* = 18)		Women right-sided surgery (*n* = 20)		Men right-sided surgery (*n* = 22)	
	Pre-surgery	Post-surgery	Pre-surgery	Post-surgery	Pre-surgery	Post-surgery	Pre-surgery	Post-surgery
Epitrack								
Interference test	21.72 ± 9.08	23.54 ± 18.31	18.72 ± 5.48	18.28 ± 5.67	19.62 ± 6.19	19.20 ± 4.82	20.62 ± 7.85	19.18 ± 5.03
TMT A	42.00 ± 25.09	56.81 ± 100.83	33.67 ± 23.09	36.39 ± 27.23	32.94 ± 18.42	36.35 ± 25.78	40.90 ± 24.01	36.32 ± 17.33
TMT B	92.48 ± 62.59	109.17 ± 118.78	80.61 ± 42.89	96.67 ± 68.89	91.63 ± 84.82	82.45 ± 36.71	115.57 ± 75.39	84.18 ± 37.77
Maze test	41.12 ± 32.57	46.19 ± 81.48	28.94 ± 17.56	23.22 ± 6.84	29.88 ± 15.88	34.65 ± 19.90	31.24 ± 16.21	43.05 ± 59.52
Word fluency	19.92 ± 8.10	21.65 ± 8.46	19.78 ± 11.49	19.22 ± 10.00	19.06 ± 8.88	20.05 ± 9.77	20.00 ± 9.18	22.41 ± 9.02
Digit span backward	4.08 ± 1.32	3.88 ± 1.34	4.17 ± 1.20	4.44 ± 1.42	4.25 ± 0.93	4.45 ± 1.40	4.38 ± 1.47	4.18 ± 1.33
Total score	30.68 ± 6.01	31.23 ± 6.35	31.56 ± 5.72	32.28 ± 6.31	32.00 ± 4.26	31.55 ± 5.65	30.57 ± 6.92	31.59 ± 6.15
WCST								
Number of errors	34.04 ± 26.51	34.42 ± 28.96	29.56 ± 21.31	33.83 ± 19.86	31.15 ± 21.60	33.20 ± 25.68	44.14 ± 26.26	45.41 ± 31.01
Number of perseverative responses	23.77 ± 28.89	20.43 ± 25.35	22.78 ± 22.26	19.22 ± 14.16	23.75 ± 22.66	24.32 ± 23.68	28.82 ± 23.74	24.81 ± 21.66
Number of non-perseverative errors	12.73 ± 11.83	15.81 ± 15.99	9.11 ± 6.01	16.28 ± 12.60	10.35 ± 6.25	12.30 ± 10.61	17.41 ± 13.00	20.68 ± 18.22
Trials to complete the first category	22.58 ± 31.62	23.92 ± 33.35	11.83 ± 2.71	15.89 ± 13.42	19.45 ± 26.33	15.15 ± 13.09	23.36 ± 27.59	41.36 ± 44.15
Failure to maintain a set	0.85 ± 1.16	0.58 ± 0.86	0.61 ± 1.04	1.00 ± 1.50	0.75 ± 0.97	0.80 ± 1.28	1.23 ± 0.97	0.91 ± 0.87
Correct responses	68.42 ± 15.42	64.81 ± 12.26	70.33 ± 10.27	72.72 ± 13.25	72.35 ± 11.73	68.05 ± 12.02	68.73 ± 14.35	61.68 ± 11.33
Conceptual level responses	60.08 ± 20.04	53.13 ± 18.69	61.00 ± 12.45	62.78 ± 14.40	60.85 ± 15.27	59.45 ± 13.63	53.86 ± 17.71	48.05 ± 19.23
Categories completed	4.88 ± 1.93	4.54 ± 2.21	5.39 ± 1.29	5.00 ± 1.37	5.20 ± 1.51	4.70 ± 1.59	3.86 ± 1.93	3.64 ± 2.34
Learning to learn	-2.32 ± 8.13	-5.73 ± 9.85	-3.51 ± 8.19	-6.95 ± 15.92	-2.51 ± 3.65	-5.29 ± 8.04	-9.45 ± 9.32	-10.50 ± 30.25
BNT	45.19 ± 6.63	39.58 ± 10.60	47.50 ± 9.53	43.33 ± 11.88	49.70 ± 7.14	50.60 ± 7.59	47.86 ± 8.70	48.82 ± 8.47
FAS	32.92 ± 11.57	34.85 ± 12.88	31.44 ± 15.95	32.44 ± 15.41	29.45 ± 11.43	31.75 ± 10.69	31.36 ± 14.08	33.41 ± 14.40
Animal Naming Test	16.00 ± 4.96	14.23 ± 5.81	17.33 ± 5.41	18.56 ± 6.91	18.60 ± 6.13	16.20 ± 6.07	18.23 ± 5.72	18.50 ± 5.33
WMS-III								
Immediate auditory memory	13.62 ± 5.02	12.00 ± 5.24	15.39 ± 5.28	13.33 ± 6.50	20.30 ± 7.41	18.10 ± 7.10	16.36 ± 4.88	16.36 ± 5.59
Immediate visual memory	14.88 ± 4.68	15.42 ± 5.67	16.11 ± 5.67	16.06 ± 5.27	18.10 ± 5.40	16.00 ± 4.69	14.73 ± 5.90	14.68 ± 5.33
Immediate memory	28.46 ± 8.65	27.35 ± 10.28	31.39 ± 10.31	29.83 ± 10.46	38.45 ± 11.17	34.45 ± 10.25	30.64 ± 9.22	31.09 ± 9.04
Delayed auditory memory	14.19 ± 4.84	12.31 ± 5.90	16.11 ± 5.85	13.72 ± 5.27	20.25 ± 6.15	19.65 ± 6.35	16.27 ± 4.70	17.09 ± 5.11
Delayed visual memory	15.50 ± 4.87	16.58 ± 5.75	16.72 ± 5.11	17.33 ± 3.63	18.25 ± 6.09	16.85 ± 5.16	14.50 ± 5.65	15.18 ± 4.16
Delayed memory	37.88 ± 10.48	34.42 ± 13.02	41.50 ± 13.01	37.67 ± 10.98	49.60 ± 17.56	44.65 ± 15.02	39.00 ± 11.69	39.64 ± 12.14
Delayed auditory recognition	8.00 ± 3.19	6.88 ± 3.78	8.67 ± 3.34	7.50 ± 3.70	10.35 ± 4.26	9.45 ± 3.32	8.23 ± 3.41	8.77 ± 2.76
STAI	27.00 ± 10.56	24.04 ± 10.96	24.56 ± 13.48	27.39 ± 16.31	22.00 ± 9.56	18.70 ± 12.57	28.00 ± 8.14	20.68 ± 11.13
BDI-II	12.27 ± 9.99	13.38 ± 12.33	12.50 ± 12.02	13.11 ± 13.74	9.90 ± 7.40	8.30 ± 10.19	13.86 ± 9.08	9.64 ± 9.56
QOLIE-31								
Seizure worry	46.21 ± 22.35	68.27 ± 22.98	52.43 ± 25.01	67.79 ± 28.16	48.90 ± 26.62	67.56 ± 30.15	51.51 ± 26.05	74.86 ± 24.62
Overall QOL	61.38 ± 17.85	76.66 ± 17.18	67.56 ± 14.36	70.19 ± 17.02	64.67 ± 15.67	67.97 ± 11.20	61.09 ± 15.56	73.32 ± 21.85
Emotional well-being	59.85 ± 19.48	61.69 ± 21.53	62.61 ± 21.31	66.22 ± 24.32	68.70 ± 16.30	70.74 ± 15.44	58.82 ± 16.20	63.36 ± 24.76
Energy	51.08 ± 20.95	61.15 ± 18.56	62.00 ± 16.51	56.67 ± 28.08	60.25 ± 15.77	60.00 ± 16.91	66.59 ± 15.54	69.23 ± 22.27
Cognition self-rating	47.12 ± 21.08	56.91 ± 22.17	54.69 ± 26.76	51.60 ± 29.84	46.91 ± 21.13	59.66 ± 26.44	45.38 ± 18.72	61.75 ± 24.48
Medication effects	45.04 ± 32.83	60.54 ± 31.58	50.47 ± 29.01	55.59 ± 35.96	40.09 ± 24.82	65.54 ± 26.62	48.18 ± 30.53	57.55 ± 29.08
Social functioning	48.62 ± 19.28	64.12 ± 21.94	57.33 ± 18.81	58.44 ± 31.50	42.85 ± 26.71	62.26 ± 25.53	47.68 ± 24.88	66.27 ± 24.89
QOLIE-31 total score	51.21 ± 14.44	61.24 ± 14.61	58.88 ± 16.13	57.74 ± 24.09	51.72 ± 15.11	64.26 ± 17.75	53.33 ± 14.06	66.33 ± 19.87

Note: BDI: Beck Depression Inventory-II; BNT: Boston Naming Test; QOL: quality of life; QOLIE-31: Quality of Life in Epilepsy Inventory; STAI: State-Trait Anxiety Inventory; TMT: Trail Making Test; WCST: Wisconsin Card Sorting Test; WMS-III: Wechsler Memory Scale-Third Edition

**Fig. 1:**
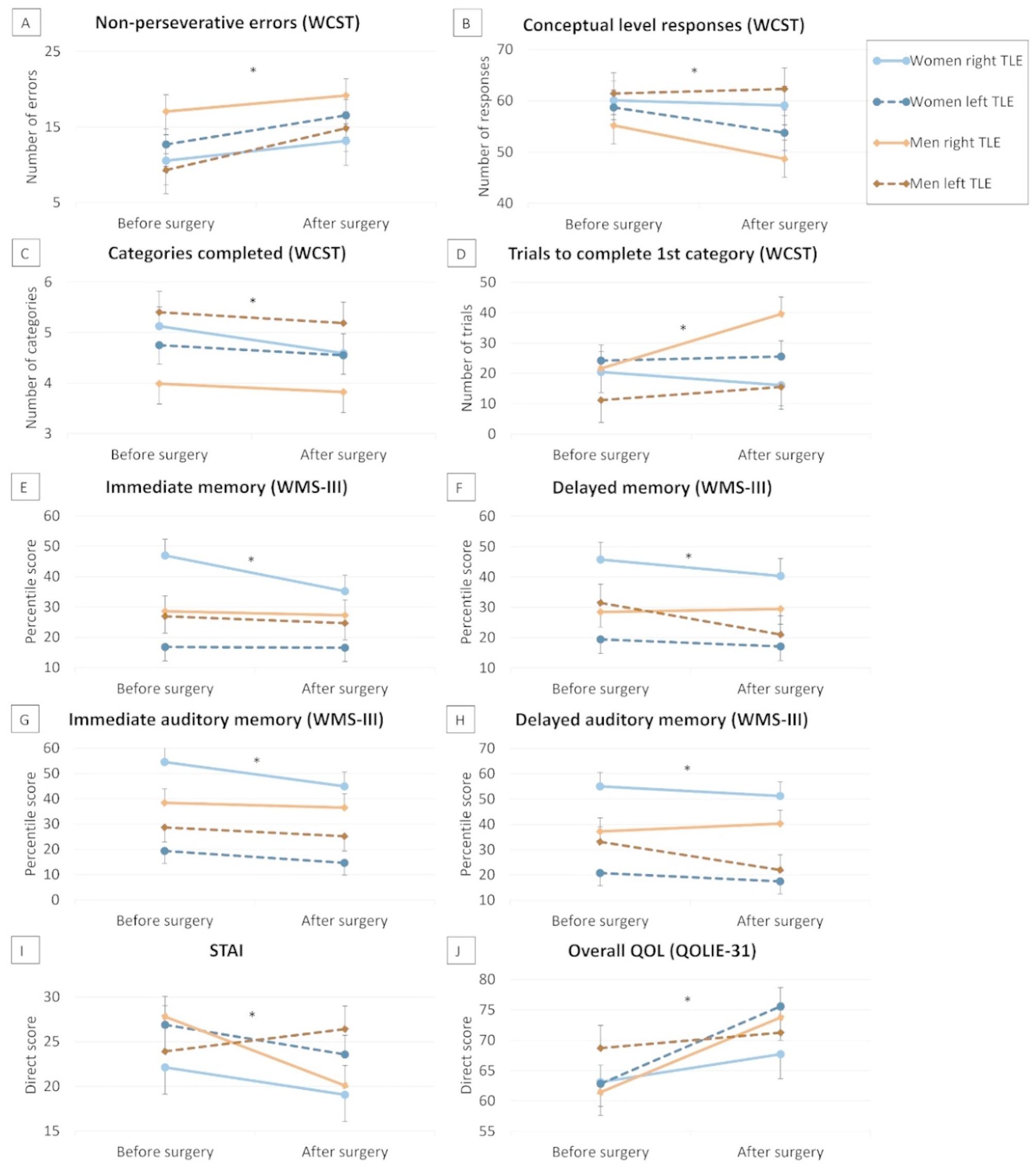
Gender and side of surgery interaction effects on cognitive functioning, affectivity and QOL. *Note*: QOLIE-31: Quality of Life in Epilepsy Inventory; STAI: State-Trait Anxiety Inventory; WCST: Wisconsin Card Sorting Test; WMS-III: Wechsler Memory Scale-Third Edition; *: p < 0.05. Statistics are adjusted by total ASM drug load. For sections **A**, **D**, and **I**, an increase in score indicates worsening, whereas for sections **B**, **C**, **E**, **F**, **G**, **H**, and **J**, an increase in score indicates improvement

The significant effects of the interaction between gender and side of surgery on cognition, affectivity, and QOL are shown in Fig. 1. Significant effects of ‘gender×side of surgery’ were found on correct responses, number of non-perseverative errors, conceptual level responses, categories completed, and trials to complete the first category of WCST (*F*[1,81] = 3.87, *p* = 0.05, η² = 0.05; *F*[1,81] = 3.95, *p* = 0.05, η² = 0.05; *F*[1,81] = 4.05, *p* = 0.048, η² = 0.05; *F*[1,81] = 5.18, *p* = 0.026, η² = 0.06; and *F*[1,81] = 5.15, *p* = 0.026, η² = 0.06, respectively). After applying Bonferroni corrections, the post hoc effect for correct responses did not remain significant, while the ‘gender×side of surgery’ effects on the number of non-perseverative errors, conceptual level responses, categories completed, and trials to complete the first category were maintained. Specifically, in the right-side surgery group, men had significantly more non-perseverative errors than women (*p* = 0.05), independently of the time point, while no gender differences were observed in the left-side surgery group (*p* = 0.42). For conceptual level responses, categories completed, and trials to complete the first category, men with left-side surgery had better performance than men with right-side surgery (for all, *p* < 0.04), independently of the time point, without differences in women depending on the side of surgery (for all, *p* > 0.46). A significant effect of ‘time point×gender’ was also found on trials to complete the first category of WCST (*F*[1,81] = 3.53, *p* = 0.05, η² = 0.04), with men having a significant postsurgical decline in their performance (*p* = 0.025) and without postsurgical changes in women (*p* = 0.75).

Significant effects of ‘gender×side of surgery’ were also found for immediate and delayed memory (i.e., general indices including both verbal and visual material), and immediate auditory and delayed auditory memory (*F*[1,81] = 5.89, *p* = 0.018, η² = 0.07; *F*[1,81] = 5.03, *p* = 0.028, η² = 0.06; *F*[1,81] = 5.09, *p* = 0.027, η² = 0.06; and *F*[1,81] = 5.13, *p* = 0.026, η² = 0.06, respectively). Specifically, women with left-side surgery had poorer performance in these variables than women with right-side surgery (for all, *p* < 0.001), without significant differences depending on the side of surgery in men (for all, *p* > 0.13). In patients with right-side surgery, men had significantly poorer immediate, delayed, and delayed auditory memory than women (for both, *p* < 0.048), without differences depending on gender in patients with left-side surgery (for both, *p* > 0.17). No significant ‘gender×side of surgery’ effects were found on immediate and delayed visual memory (*F*[1,81] = 1.77, *p* = 0.19, η² = 0.02; and *F*[1,81] = 2.57, *p* = 0.11, η² = 0.03, respectively). Significant main effects of ‘side of surgery’ were found on immediate, delayed, immediate auditory and delayed auditory memory, and delayed auditory recognition (*F*[1,81] = 8.34, *p* = 0.005, η² = 0.10; *F*[1,81] = 7.81, *p* = 0.007, η² = 0.09; *F*[1,81] = 19.29, *p* < 0.001, η² = 0.20; *F*[1,81] = 20.17, *p* < 0.001, η² = 0.20; and *F*[1,81] = 3.85, *p* = 0.05, η² = 0.05, respectively), with patients with right-side surgery having better performance than those with left-side surgery, independently of the time point.

Regarding affectivity, a significant effect of ‘time point×gender×side of surgery’ was found on STAI (*F*[1,81] = 5.13, *p* = 0.026, η² = 0.06). Specifically, men with right-side surgery had a reduction in anxiety scores after surgery (*p* < 0.001), without significant postsurgical changes in the rest of the groups. A significant effect of ‘time point×side of surgery’ was also found on STAI (*F*[1,81] = 4.64, *p* = 0.03, η² = 0.06), with a significant reduction in anxiety scores after surgery in patients with right-side surgery (*p* < 0.001), but no postsurgical changes in patients with left-side surgery (*p* = 0.80).

For QOL, a significant effect of ‘time point×gender×side of surgery’ was found on overall QOL (*F*[1,81] = 4.50, *p* = 0.037, η² = 0.06). Specifically, women with left-side surgery and men with right-side surgery had improvements in QOL after surgery (for both, *p* < 0.004), without postsurgical changes in the other groups (for both, *p* > 0.30). Furthermore, significant effects of ‘gender’ were found on the energy subscale (*F*[1,81] = 3.84, *p* = 0.05, η² = 0.05), with men having better QOL than women. Significant effects of ‘time point×side of surgery’ were also found on cognitive self-assessment subscale and total QOL score (*F*[1,81] = 4.37, *p* = 0.04, η² = 0.05; *F*[1,81] = 4.48, *p* = 0.038, η² = 0.06, respectively), with patients with right-side surgery having post-surgical improvements (for both, *p* < 0.001), without postsurgical changes in left-sided surgery patients (for both, *p* > 0.13).

No significant effects of the interaction between gender and side of surgery were found on other cognitive variables. However, isolated effects of gender or side of surgery were found on semantic fluency, attention, and naming. Specifically, significant effects of ‘gender’ and ‘time point×gender’ were found for semantic fluency (*F*[1,81] = 4.65, *p* = 0.03, η² = 0.06; and *F*[1,81] = 5.31, *p* = 0.024, η² = 0.06, respectively), with women having poorer scores than men (*p* = 0.03) and a significant decline after surgery (*p* = 0.006), and men having no significant postsurgical changes (*p* = 0.56). Furthermore, a significant effect of ‘time point×side of surgery’ was found on the TMTA subtest of Epitrack (*F*[1,81] = 6.88, *p* = 0.01, η² = 0.09), with patients with right side surgery having a significant improvement after surgery (*p* = 0.046), without changes after surgery in the group with left-side surgery (*p* = 0.10). Significant effects of ‘side of surgery’ and ‘time point×side of surgery’ were also found on BNT (*F*[1,81] = 11.98, *p* < 0.001, η² = 0.13; and *F*[1,81] = 17.33, *p* < 0.001, η² = 0.18, respectively). Specifically, patients with left-side surgery had poorer performance than those with right-side surgery (*p* < 0.001) and showed a significant decline after surgery (*p* < 0.001), whereas those with right side of seizure had no postsurgical changes (*p* = 0.34).

**Fig. 2:**
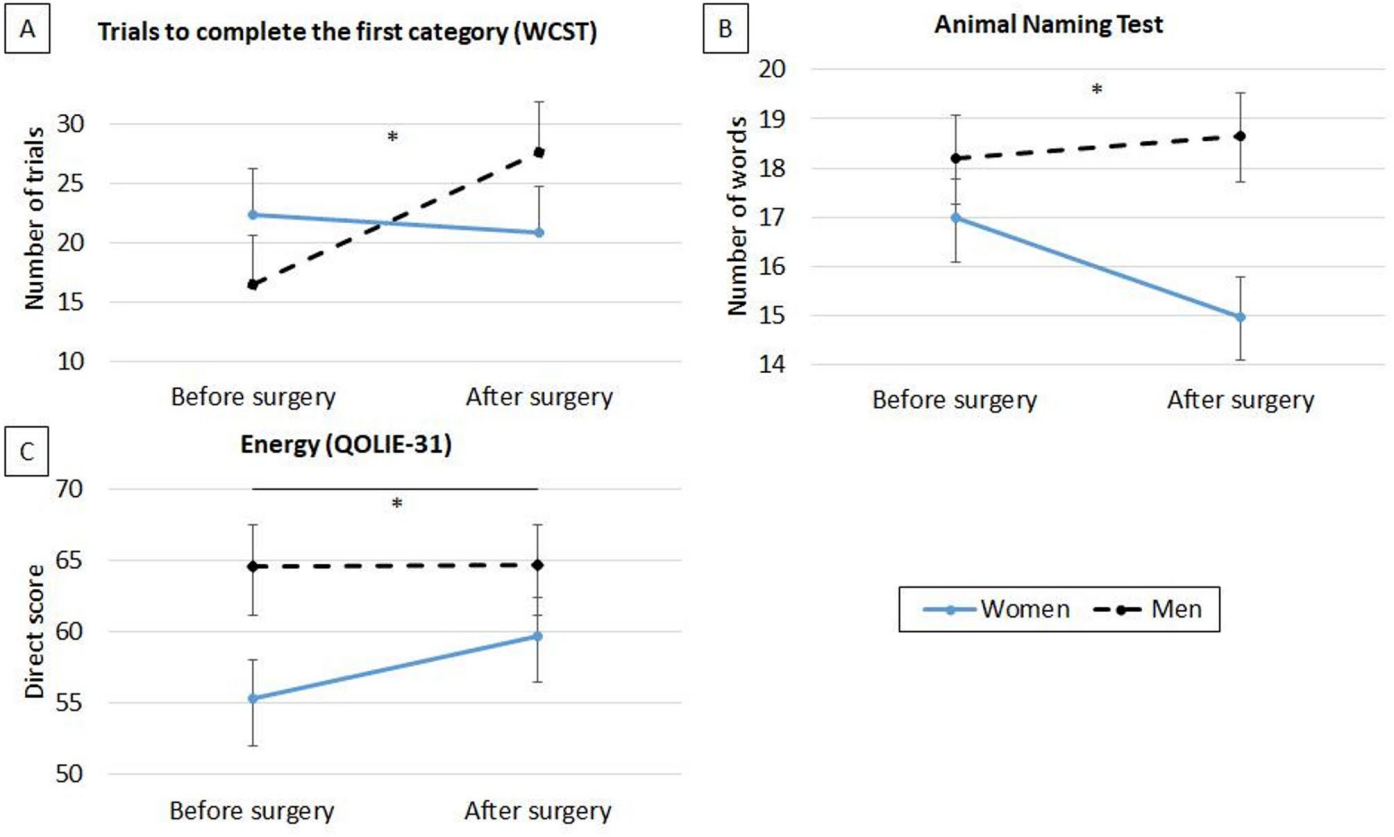
Gender effects on cognitive functioning and QOL. *Note*: QOLIE-31: Quality of Life in Epilepsy Inventory; WCST: Wisconsin Card Sorting Test; *: p < 0.05. Statistics are adjusted by total ASM drug load. For sections **B** and **C**, an increase in score indicates improvement, whereas for section **A**, an increase in score indicates worsening

**Fig. 3:**
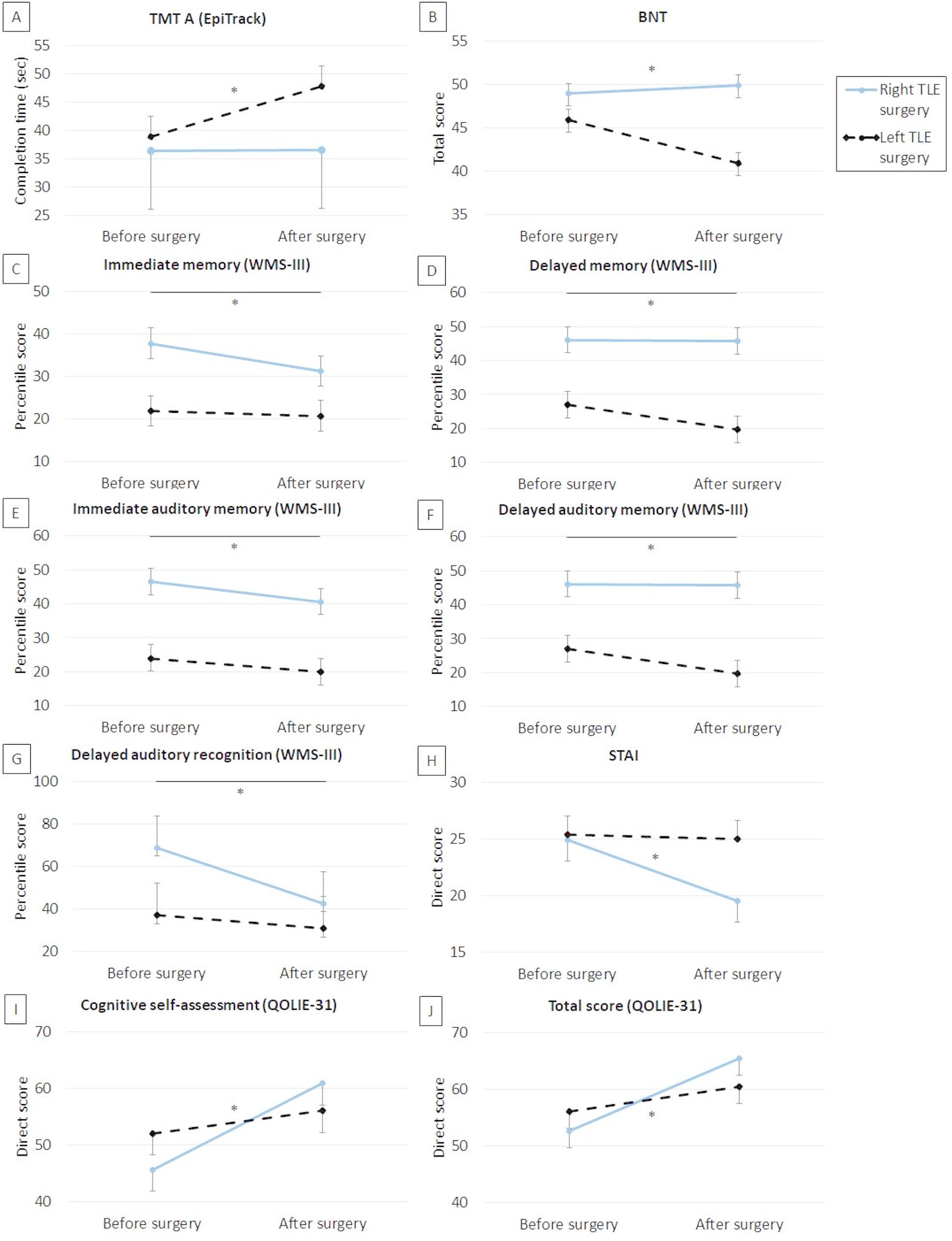
Side of surgery effects on cognitive functioning, affectivity and QOL. *Note*: BNT: Boston Naming Test; QOLIE-31: Quality of Life in Epilepsy Inventory; STAI: State-Trait Anxiety Inventory; WMS-III: Wechsler Memory Scale-Third Edition; *: p < 0.05. Statistics are adjusted by total ASM drug load. For sections **B**, **C**, **D**, **E**, **F**, **G**, **I**, and **J**, an increase in score indicates improvement, whereas for sections **A** and **H**, an increase in score indicates worsening

The main effects of gender and side of surgery are shown in Figs. 2 and 3, respectively. No other significant effects were found.

## Discussion

The present study reveals that gender is a significant modulator of cognitive functioning, affectivity, and QOL, and interacts with the side of surgery in patients with TLE undergoing surgery. In the group with right-side surgery, men had poorer executive functioning and memory than women. Furthermore, men with right-side surgery showed poorer executive function than those with left-side surgery, and a decrease in anxiety levels following surgery. Women with right-side surgery had better memory than those with left-side surgery. Both women and men had postsurgical QOL improvements modulated by the side of surgery. Independently of the side of surgery, women had poorer semantic fluency and QOL than men and a significant postsurgical decline in semantic fluency, whereas men had a significant postsurgical decline in executive functions.

Surgery was successful in the control of seizures one year after surgery, as 84.1% of the patients were seizure-free. Most of the patients did not experience meaningful cognitive changes after surgery, although it should be noted that 27.9% showed clinically significant improvements in word fluency (EpiTrack), 27.9% had a clinically significant decline in naming, and 10.5% had a significant decline in delayed memory. Regarding affectivity and QOL, results were more variable, 25.6% had a decrease in anxiety scores and 41.9% showed QOL improvements.

IQ did not vary depending on gender, side of seizure focus or their interaction. However, significant differences emerged in specific cognitive domains. In the group with right TLE, men performed worse in executive functions than women, independently of the time point (pre- or post-surgery). Furthermore, men showed a significant postsurgical decline in cognitive flexibility, regardless of the side of surgery, with small effect sizes, while women experienced no significant changes. To our knowledge, this is the first study to examine gender differences in changes to attention and executive functions after TLE surgery. Cairós-González et al. [12] suggested that the right temporal lobe plays a critical role in cognitive flexibility, which relies on extensive bidirectional connections with frontal regions. Our findings suggest that men’s neural networks for executive functions may be especially vulnerable. Men generally exhibit a more lateralized brain organization, whereas women show stronger interhemispheric connectivity [41]. This difference could explain, at least in part, our findings, as enhanced bilateral engagement could help women better compensate when one hemisphere is impaired [6], whereas men’s reduced interhemispheric connectivity may increase their susceptibility to executive dysfunction, particularly after right-sided disruption. Furthermore, in the group of men, patients with right TLE had poorer executive function than those with left TLE. These results align with Cairós-González et al. [12] and Corcoran and Upton [13], who examined the role of side of seizure focus without considering gender. However, they differ from Black et al. [11] and Tudesco et al. [14], likely due to differences in epilepsy types [11] and the cognitive tests used [14].

In the group with right TLE, men showed significantly poorer memory than women, with medium effect sizes, independently of the time point (pre- or post-surgery). These differences emerged in verbal memory and in the general memory indices (i.e., composite indices combining verbal and visual subtests), but not in the specific visual memory indices. The null findings for visual memory may partly reflect the limited construct validity and lateralizing value of visual memory subtests such as Family Pictures, which has been shown to load more heavily on verbal memory [42] and was therefore removed from WMS-IV. Consequently, our findings suggest that the observed gender-by-side of surgery effects on memory were primarily driven by verbal memory performance, with significant effects on the composite scores likely reflecting the conflation of material-specific memory processes within these indices. No gender-related differences were found in left TLE patients. These results align with previous research indicating superior verbal memory in women, although this difference has been reported independently of seizure focus [15, 16]. Our findings suggest that being a woman may serve as a protective factor for verbal memory function, specifically in right TLE, though not due to overall verbal abilities, as women performed worse in the semantic fluency task. Baxendale et al. [19] also observed better verbal memory function in women despite a broader impairment of verbal abilities, suggesting greater interhemispheric reorganization of memory in the female brain. Despite this advantage, we found no gender differences in postsurgical memory changes. Given that better presurgical memory reflects hippocampal integrity - left for verbal memory and right for visual memory [43]- patients with right TLE should theoretically experience memory improvements if left hippocampal integrity is preserved (functional reserve theory) [44] or memory declines if right hippocampal integrity is high (functional adequacy theory) [45]. Since women with right TLE performed better on general and verbal memory indices, these opposing mechanisms may have balanced each other, explaining the absence of gender-by-side of surgery effects on postsurgical memory outcomes.

As indicated above, in the semantic fluency task, women performed worse than men and exhibited a significant decline after surgery, regardless of the side of surgery, with medium effect sizes. As far as we know, only Eichstaedt et al. [46] examined this issue, and found a lack of significant main effects of gender, despite an interaction between gender and side of seizure focus. In contrast, studies in the general population suggest a female advantage in verbal abilities, though these differences may be marginal [47]. Our findings may be influenced by the low reliability of the test (test-retest reliability = 0.56), so the semantic fluency postsurgical changes derived from RCI should be interpreted with caution. Nevertheless, this low reliability may affect both genders equally, so these findings cannot be attributed solely to measurement reliability. A further explanation may be related to the nature of the semantic fluency task, as no gender differences were observed in other verbal abilities, such as verbal IQ, naming, or phonemic fluency. Semantic fluency depends on the organization of words within the mental lexicon, requiring efficient executive control to retrieve words from different subcategories [48]. The hunter-gatherer hypothesis posits that this organization is shaped by social roles during development [47], which may explain findings of a male advantage in the “animal” category in healthy participants [49]. Furthermore, surgery may differentially affect semantic verbal fluency networks depending on gender. Women with TLE often demonstrate a more bilateral representation of language than men [6], which may offer some protection against general verbal declines. However, semantic fluency relies on intact frontotemporal connectivity, and surgical disruption of these networks may limit full functional reorganization [50].

Men who underwent right-sided surgery showed a post-surgical decrease in anxiety levels, with medium effect sizes. In contrast, Baxendale et al. [19] found no significant gender differences in affectivity or its interaction with the side of surgery. This discrepancy could be due to the different instruments used to assess anxiety, since we evaluate trait anxiety instead of clinical anxiety. Both men and women experienced postsurgical QOL improvements modulated by the side of surgery (with medium effect sizes), with significant QOL improvements in women with left-side surgery and men with right-side surgery. Notably, despite poorer executive functioning and memory, men with right-sided surgery still reported greater affective and QOL benefits post-surgery. This may be partly explained by the unique neurobiological and psychosocial challenges faced by women with epilepsy, including the impact of hormonal fluctuations on seizure frequency, contraception, and potential drug-drug interactions [51]. Furthermore, socially marked behaviors associated with gender roles may influence how individuals perceive and adapt to cognitive challenges and condition their QOL [8]. Women are often more likely to report cognitive or affective difficulties, partly due to social expectations, while men may underreport such challenges in line with cultural norms of stoicism and independence [52]. In addition, women are more frequently in caregiving positions than men [53], which can contribute to poorer physical recovery and greater fatigue following surgery. Consistent with this, in our study, men had higher perceived levels of energy than women, with small effect sizes. Previous studies have also found that the female gender is associated with poor QOL in patients with epilepsy [54–56], without exploring cognitive and affective variables.

Regarding the side of surgery, patients with left-side surgery performed worse in naming and memory compared to right-sided surgery patients, with medium to large effect sizes, and showed a postsurgical naming decline, consistent with prior studies [4, 9]. Furthermore, patients with right-side surgery showed a significant postsurgical improvement in attention and executive function (i.e., TMT-A subtest of Epitrack), in line with the nociferous cortex hypothesis, which posits that surgical removal of the epileptogenic focus in TLE may reduce its disruptive influence on extratemporal networks, thereby facilitating executive function improvements [10]. Additionally, our results show that right-sided surgery patients experienced postsurgical reductions in anxiety and improvements in QOL, with medium effect sizes, without significant changes in left-sided surgery patients. Andelman et al. [57] also reported lower anxiety and higher QOL in right TLE patients, suggesting that elevated anxiety may be a surgery-related risk factor. Overall, our findings suggest that patients undergoing right-sided surgery show better neuropsychological outcomes compared to those with left-sided surgery, which may be related to the observed reductions in anxiety and improvements in QOL in this group.

This study has strengths such as the relatively homogeneous sample, the comprehensive assessment of different cognitive domains, and, as far as we know, it is the first to explore the effects of gender and its interaction with the side of surgery in cognition, affectivity, and QOL (considering these variables jointly) in patients undergoing TLE surgery. However, some limitations should be noted. First, larger sample sizes could provide more information about groups and ensure statistical power. Second, although most patients in the total sample underwent anterior temporal lobectomy with amygdalohippocampectomy, there was some variability in surgical approaches across subgroups -particularly among men with left-sided surgeries, where about half underwent temporal lobe lesionectomies. While these differences were not statistically significant, this imbalance may have influenced cognitive outcomes. The extent of lesionectomies varied depending on lesion location and surgical planning, and, in particular, cortical lesionectomies are considered more selective procedures and have been associated with more favorable cognitive outcomes than temporal lobectomies [58]. Third, the inclusion of multiple WCST indices to capture a comprehensive profile of executive functioning may have inflated Type 1 error, so we applied Greenhouse-Geisser corrections and Bonferroni adjustments to minimize this limitation. Fourth, postsurgical cognitive changes were assessed at a single time point, underscoring the need for longitudinal studies that examine long-term trajectories. Fifth, the proportions of improvement, stability, and decline based on RCI should be interpreted with caution, since, in most cases (except for Epitrack), RCIs were derived from normative data from the general population, which may narrow the cut-off bands and consequently overestimate rates of cognitive decline. The development of epilepsy-specific normative data is recommended. Sixth, future research should explore additional factors such as gender roles and sexual hormones. Finally, although QOL instruments provide a comprehensive view of the impact of illness and treatment [59], they rely on patient perceptions, which may not fully align with their actual functionality in daily life.

### Perspectives and significance

Clinically, our results underscore the need for gender-specific targeted cognitive and affective support. For men, post-surgical cognitive interventions focusing on executive function and verbal memory may be particularly beneficial, especially after right-sided surgeries. For women, strategies to support and maintain semantic fluency are recommended. Additionally, incorporating anxiety and QOL support into postsurgical care is crucial to optimize outcomes – with individualized approaches that account for gender and side of surgery.

## Conclusion

Our findings highlight the importance of considering both gender and the side of surgery in understanding cognitive, affective, and QOL outcomes in patients with TLE undergoing surgery. Women are at higher risk of semantic fluency deficits and declines following surgery, while men show greater vulnerability to deficits in verbal memory and executive function, particularly following right-sided surgery, as well as postsurgical declines in executive functioning.

## Data Availability

The datasets used and/or analyzed during the current study are available from the corresponding author on reasonable request.
